# Incidence and persistence of carcinogenic genital human papillomavirus infections in young women with or without *Chlamydia trachomatis* co-infection

**DOI:** 10.1002/cam4.496

**Published:** 2015-07-21

**Authors:** Henrike J Vriend, Johannes A Bogaards, Jan E A M van Bergen, Antoinette A T P Brink, Ingrid V F van den Broek, Christian J P A Hoebe, Audrey J King, Marianne A B van der Sande, Petra F G Wolffs, Hester E de Melker

**Affiliations:** 1 National Institute for Public Health and the Environment (RIVM), Centre for Infectious Disease ControlBilthoven, The Netherlands; 2 Division of Infectious Diseases, Department of Internal Medicine, Center for Infection and Immunity Amsterdam (CINIMA), Academic Medical Center (AMC)Amsterdam, The Netherlands; 3 STI AIDS NetherlandsAmsterdam, The Netherlands; 4 Department of General Practices, Academic Medical Centre, University of AmsterdamAmsterdam, The Netherlands; 5 Department of Medical Microbiology, Maastricht University Medical Center (MUMC)Maastricht, The Netherlands; 6 South Limburg Public Health ServiceGeleen, The Netherlands; 7 Julius Center, University Medical CenterUtrecht, The Netherlands

**Keywords:** Cervical cancer, *Chlamydia trachomatis*, human papillomavirus, sexually transmitted infection, viral infection

## Abstract

We assessed whether infection with chlamydia increases the incidence of carcinogenic human papillomavirus (HPV) infections and if HPV persistence is affected by chlamydia co-infection. For 1982 women (16–29 years-old) participating in two consecutive rounds of a chlamydia screening implementation trial, swabs were polymerase chain reaction tested to detect chlamydia and 14 carcinogenic HPV genotypes. HPV type-specific incidence and persistence rates were stratified for chlamydia positivity at follow-up. Associations were assessed by multilevel logistic regression analyses with correction for sexual risk factors. HPV type-specific incidence ranged from 1.4% to 8.9% and persistence from 22.7% to 59.4% after a median follow-up of 11 months (interquartile range: 11–12). Differences in 1-year HPV persistence rates between chlamydia -infected and noninfected women were less distinct than differences in HPV incidence rates (pooled adjusted odds ratios of 1.17 [95% CI: 0.69–1.96] and 1.84 [95% CI: 1.36–2.47], respectively). The effect of chlamydia co-infection on HPV-infection risk did not significantly differ by HPV genotype. In conclusion, infection with chlamydia increases the risk of infection by carcinogenic HPV types and may enhance persistence of some HPV types. Although these findings could reflect residual confounding through unobserved risk factors, our results do give reason to explore more fully the association between chlamydia and HPV type-specific acquisition and persistence.

## Introduction

Cervical carcinoma is the third most common cancer among women worldwide, and most cases occur in developing countries 1. Persistent infection with carcinogenic human papillomavirus (HPV), a highly prevalent sexually transmitted infection (STI), is a necessary cause of cervical carcinoma. The carcinogenic process following HPV infection is likely influenced by behavioral and biological cofactors. One possible cofactor is *Chlamydia trachomatis* (hereinafter, chlamydia) infection, an STI that often remains asymptomatic. The implication of chlamydia in the pathogenesis of cervical carcinoma is controversial, as its interference has not been demonstrated at a particular stage of the carcinogenic process. Alternatively, chlamydia could increase susceptibility to HPV co-infection 2 or decrease the efficient clearance of an existing HPV infection, thereby increasing the chance of developing cancer 3–5.

The presence of chlamydia antibodies has been associated with an increased risk for invasive squamous cell carcinoma of the cervix, although no association was seen with adeno- or adenosquamous cell carcinoma 6–8. In addition, no association was found for the effect of a chlamydia co-infection on the presence of high-grade cervical intraepithelial neoplasia (CIN2+) 2,9.

Epidemiological studies on the impact of chlamydia on HPV acquisition and/or persistence have yielded equivocal results due to the difficulty in separating biological from behavioral effects. If a biological association exists, it might differ among HPV types, which differ in clearance time 10 and carcinogenic potential 11. To explore the possible association of chlamydia infection with different HPV types, we analyzed type-specific incidence and persistence of HPV by chlamydia positivity in a prospective cohort study among young sexually active women, correcting for known risk factors to assess the association of HPV with chlamydia independent of sexual behavior.

## Materials and Methods

### Study design

The study design is detailed elsewhere 12,13. Briefly, between 2008 and 2012 a systematic, selective, Internet-based Chlamydia Screening Implementation (CSI) program was rolled out in three areas in the Netherlands. Based on population registers, invitation letters were sent annually to all 16–29-year-old women of Amsterdam, Rotterdam, and selected municipalities of South Limburg. Respondents could access a website (www.chlamydiatest.nl) with a personal code to complete a voluntary questionnaire and request a home-sampling test package. Questions addressed ethnicity, educational level, the number of sexually active years (continuous variable), number of sexual partners in the 6 months prior to follow-up measurement (continuous variable), and condom use with a steady and casual partner in the 6 months prior to follow-up measurement (categorical variable).

In total, 16,679 women participated in two or more rounds. Of participants in rounds 1 and 2, 5% tested chlamydia-positive at round 1, of whom 7% were also positive at round 2. These percentages were similar for participants in rounds 2 and 3 (pers. comm; 12). A subset of the vaginal swabs from women consenting to testing for infections other than chlamydia and participating in two rounds between 2008 and 2011 were tested for HPV infection in 2011.

The trial was approved by the Medical Ethics Committee Free University Amsterdam (Identification number 2007/239), the Netherlands.

### Chlamydia testing

Self-collected vaginal swabs were sent to three regional laboratories in an envelope suited for mailing biological materials. Participants were instructed to post the sample immediately after self-collection. The samples were tested for chlamydia within 7 days after reception of the material in the laboratory with three commercially available nucleic acid amplification tests according to manufacturers' guidelines (PCR, Roche Cobas Taqman, San Francisco, CA; SDA, Becton Dickinson ProbeTec ET system, Sparks, MD; and Aptima CT, GenProbe Tigris, San Diego, CA). One laboratory pooled five samples for testing and, if a pool tested positive, all five were retested. The two laboratories that did not pool confirmed each chlamydia-positive sample by retesting the specimen. Infections found at baseline were treated following CSI protocol; thus chlamydia infections found at follow-up were defined as newly acquired. Remaining material from the swab was prepared for future STI testing and stored in the Biobank at −80°C.

### HPV-DNA testing

The HPV testing protocol is described elsewhere 14. Briefly, we defrosted the research samples of the selected vaginal swabs. The time interval between storage and defrosting was around 2.5 years for samples collected in 2008 and a few months for samples collected in 2011. To be certain there was no material loss due to the isolation, transportation and storage, samples were first spiked with phocine herpes virus before DNA-isolation. In addition, HPV-DNA was amplified using the SPF10 primer set according to the manufacturer's instructions (DDL Diagnostic Laboratory, Rijswijk, the Netherlands 15). HPV-specific amplicons were detected using the highly sensitive DNA enzyme-linked immunoassay. Amplicons of HPV-positive samples were subsequently analyzed in line probe assay, which detects 24 carcinogenic and noncarcinogenic HPV genotypes. In this study, we included all 14 carcinogenic and probable carcinogenic genotypes as defined by the International Agency for Research on Cancer: HPV16, -18, -31, -33, -35, -39, -45, -51, -52, -53, -56, -58, -59, and -66 11. An incident HPV infection was defined as positivity for a certain HPV type at follow-up when negativity was found at baseline. HPV persistence was defined as the detection of the same HPV genotype in both rounds.

### Statistical analysis

To assess the association of chlamydia with 1-year HPV persistence, we selected all women of whom a chlamydia and HPV test result for two consecutive study rounds was available and the time between both rounds was between 8 and 16 months.

For the descriptive analysis, we calculated the percentage of incident and persistent HPV infections among women with and without a chlamydia infection. The reference group for HPV incidence was negative for a certain type at both rounds; the reference group for HPV persistence was being positive for a certain type at baseline but negative for that type at follow-up. Note that each person was included in either analysis, but not both, for a particular HPV type. Due to the type-specific reference groups, numbers differed among the HPV types with corresponding power fluctuations. The Pearson chi-square test was used to compare percentages and to test if observed differences were significant, *P *<* *0.05. When numbers were smaller than five, Fisher's exact test was used instead of the chi-square test.

Type-specific and pooled associations between HPV incidence or persistence and chlamydia were analyzed using multilevel logistic regression models using generalized estimating equations. To model associations between HPV type-specific responses on the level of the participants, we made use of the alternating logistic regression algorithm of the SAS procedure GENMOD, assuming an exchangeable correlation structure between pairs of responses 16. Because a person could belong to distinct groups per HPV type (i.e., incident or persistent), the number of repeated measurements varied per subject. In all models, we corrected for sexual risk factors, that is, number of years sexually active, number of sexual partners in the past 6 months, condom use combined with type of sexual partner (i.e., steady and/or casual partner(s)) in the past 6 months, all at follow-up. We considered those variables only at follow-up, as we assumed they were most relevant to a newly acquired HPV infection. Given the protocol to treat chlamydia infections shortly after detection, we expected such infections at baseline to have little or no effect on HPV acquisition or persistence. Therefore, we focused on chlamydia infection at follow-up. As a comparison, however, we estimated the adjusted odds ratio (AOR) of chlamydia infection at baseline on HPV acquisition and persistence.

As not all participants completed a questionnaire, sexual risk variables were missing for about 25% of women at follow-up (Table1). Taking data from baseline measurements as a proxy for follow-up would be inappropriate, as a positive STI test result could influence sexual risk behavior 17,18. To make efficient use of the data and minimize imprecise or biased estimates of associations, we imputed the missing values related to sexual risk factors by using complete cases, that is, women who completed the questionnaire. Missing data were imputed 20 times in R 19, using the mice package 20. To achieve convergence, one participant reporting an exceptionally high number of sexual partners (≥50 partners in the past 6 months) was excluded prior to imputation. Each imputed dataset was analyzed by standard complete data procedures which ignored the distinction between real and imputed values. The results of the analyses were combined using PROC MIANALYZE in SAS statistical package version 9.3, (SAS Institute Inc., Cary, NC, USA).

**Table 1:** Summary of the characteristics at the follow-up round for 1982 Dutch women participating in the chlamydia screening implementation study between 2008 and 2011: complete cases and imputed dataset

	Complete cases	Imputed dataset
	*n* (%)	*n* (%)
Age		
Median (IQR)	25 [22–27]	25 [22–27]
Ethnicity		
Netherlands	1430 (72.1)	1430 (72.1)
Turkey	10 (0.5)	10 (0.5)
Northern Africa (Morocco)	16 (0.8)	16 (0.8)
Surinam	129 (6.5)	129 (6.5)
Dutch Antilles	60 (3.0)	60 (3.0)
Eastern Europe	23 (1.2)	23 (1.2)
Sub-Sahara Africa	33 (1.7)	33 (1.7)
Latin America	21 (1.1)	21 (1.1)
Europe other	117 (5.9)	117 (5.9)
Asia	124 (6.3)	124 (6.3)
Northern America/Canada/Oceania	19 (1.0)	19 (1.0)
Educational level^1^		
Low	411 (20.7)	399 (20.1)
High	1571 (79.3)	1583 (79.9)
Years sexually active^2^		
Median (IQR)	8 [5–10]	8 [5–10]
Condom use with steady and casual partner in the last 6 months^2^		
Steady partner and inconsistent condom use casual partner	281 (18.7)	393 (19.8)
Steady partner and consistent condom use casual partner	65 (4.3)	92 (4.6)
No steady partner and consistent condom use casual partner	332 (22.1)	447 (22.5)
No casual partner and inconsistent condom use steady partner	594 (39.5)	767 (38.7)
No casual partner and consistent condom use steady partner, or no steady partner	231 (15.4)	284 (14.3)
Number of sexual partners last 6 months^2^		
0 partners	87 (5.8)	115 (5.8)
1 partner	901 (60.0)	1153 (58.2)
2 partners	275 (18.3)	375 (18.9)
≥3 partners	239 (15.9)	339 (17.1)
STI ever^2^		
Never tested	152 (11.1)	230 (11.6)
Tested but negative	872 (63.6)	1221 (61.6)
Tested and positive	348 (25.4)	531 (26.8)
Time between baseline and follow-up		
8–12 months	1447 (73.0)	1447 (73.0)
12–16 months	535 (27.0)	535 (27.0)

STI, sexually transmitted infection.

1 Educational level is based on baseline and follow-up. Therefore, numbers among complete cases and imputed dataset can differ.

2 Due to missing data the number of complete cases for the following characteristics are: years sexually active (*n* = 1507), condom use with steady and casual partner in the last 6 months (*n* = 1503), number of sexual partners in the last 6 months (*n* = 1503), STI ever (*n* = 1372).

## Results

### Study population characteristics

Results of chlamydia testing and HPV genotyping were available for 2051 women participating in two consecutive rounds. Of these, 1982 (97%) women were retested within the required 8- to 16-month interval with a median interval of 11 months (interquartile range [IQR]: 11–12). At follow-up the median age was 25 years (IQR: 22–27), most women were of Dutch origin and highly educated. In more than half, first sexual intercourse took place at age 17, resulting in a median number of eight sexually active years (IQR: 5–10). Sixty per cent reported having one sexual partner in the last 6 months prior to follow-up, 18% had two partners, and 16% had three or more. Characteristics are given in Table1 for both the complete cases as the imputed dataset.

### Chlamydia

Of the 1982 women, 108 (5.5%) tested positive for chlamydia at baseline, 66 (3.3%) at follow-up. Five women were chlamydia positive at both rounds. For women with and without baseline infection, the median follow-up time was similar: 11 months (IQR: 11–12).

### HPV

At baseline, 1324 (66.8%) of the 1982 women were infected with HPV. Of these, 513 women had a single HPV infection, 640 women were infected with multiple types and of 171 women the specific type could not be identified. At follow-up 1419 (71.6%) women were HPV infected of which 538 with a single HPV type, 708 with multiple HPV types and 173 with an undetectable type.

### HPV incidence

**Figure 1:**
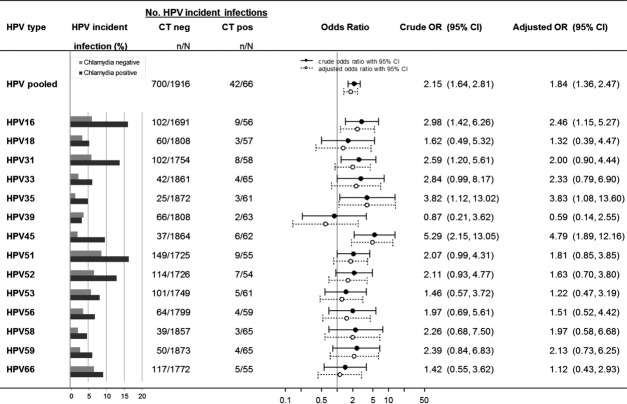
Effect of *Chlamydia trachomatis* (CT) infection at follow-up on type-specific human papillomavirus (HPV) incidence among 1982 Dutch women participating in the Chlamydia Screening Implementation study between 2008–2011. The following data is given for 14 high-risk HPV types, pooled and type-specific, using the imputed data set: number of HPV-incident cases stratified by CT positivity at follow-up; crude and adjusted odds ratio with 95% confidence intervals. The model was adjusted for number of years being sexually active, number of sexual partners in the last 6 months, condom use with the steady and/or casual partner. The denominator of the pooled estimate is the number of individuals negative for at least one of the 14 HPV types at baseline, the numerator is the number of individuals with at least one incident infection. For the type-specific estimates, the denominator is the number of individuals negative for the specific HPV type at baseline, the numerator is the number of individuals with an incident infection for the specific HPV type.

The five most incident HPV types over the follow-up period were HPV16 (6.4%), HPV31 (6.1%), HPV51 (8.9%), HPV52 (6.8%), and HPV66 (6.7%), whereas the least incident was HPV35 (1.4%) (Table2). The average follow-up time of women with incident infections did not differ from that of the total study population (i.e., 351 vs. 352 days, respectively). When stratifying for chlamydia positivity at follow-up, more incident HPV infections were detected among chlamydia*-*positive women than among -negative women (Fig.1). The pooled incidence rate was 63.6% (95% confidence interval [CI]: 51.6–74.2) and 36.5% (95% CI: 34.4–38.7) in chlamydia-positive and chlamydia-negative women, respectively. The pooled estimate of the association of chlamydia with all HPV types was significant in both univariable and multivariable analyses. Multivariable analyses showed an AOR of 1.84 (95% CI: 1.36–2.47). The HPV type-specific AORs ranged from 0.59 to 4.79 and showed significant associations of chlamydia with HPV16 (AOR: 2.46 [95% CI: 1.15–5.27]), HPV35 (AOR: 3.83 [95% CI: 1.08–13.60]), and HPV45 (AOR: 4.79 [95% CI: 1.89–12.16]) (Fig.1). Effect estimates were slightly larger in analyses without correction for sexual risk factors. Despite the observed differences in the association of chlamydia with the 14 carcinogenic HPV types, ORs did not differ significantly from one another (*P *=* *0.29). Results from the analysis on imputed data did not differ from complete case analysis, as shown in Figure S1.

**Table 2:** Number and percentage of incident and persistent type-specific HPV infections among 1982 Dutch women participating in the chlamydia screening implementation study between 2008 and 2011, given for the complete cases and the imputed dataset

	HPV incidence				HPV persistence			
	Complete cases		Imputed dataset		Complete cases		Imputed dataset	
	*n*/*N*	% Incident	*n*/*N*	% Incident	*n*/*N*	% Persistent	*n*/*N*	% Persistent
HPV16	93/1319	7.1	111/1747	6.4	103/182	56.6	137/235	58.3
HPV18	49/1407	3.5	63/1865	3.4	41/94	43.6	50/117	42.7
HPV31	88/1361	6.5	110/1812	6.1	84/140	60.0	101/170	59.4
HPV33	34/1458	2.3	46/1926	2.4	21/43	48.8	27/56	48.2
HPV35	17/1464	1.2	28/1933	1.4	22/37	59.5	27/49	55.1
HPV39	48/1421	3.4	68/1871	3.6	31/80	38.8	42/111	37.8
HPV45	35/1461	2.4	43/1926	2.2	22/40	55.0	28/56	50.0
HPV51	125/1356	9.2	158/1780	8.9	67/145	46.2	88/202	43.6
HPV52	82/1345	6.1	121/1780	6.8	58/156	37.2	84/202	41.6
HPV53	76/1379	5.5	106/1810	5.9	53/122	43.4	73/172	42.4
HPV56	59/1404	4.2	68/1858	3.7	46/97	47.4	54/124	43.5
HPV58	32/1460	2.2	42/1922	2.2	19/41	46.3	25/60	41.7
HPV59	41/1471	2.8	54/1938	2.8	6/30	20.0	10/44	22.7
HPV66	92/1381	6.7	122/1827	6.7	43/120	35.8	51/155	32.9

HPV, human papillomavirus.

A nonsignificant AOR closer to one was found when estimating the effect of chlamydia at baseline on HPV acquisition in comparison to the effect of chlamydia at follow-up on HPV acquisition (AOR: 1.09 [95% CI: 0.82–1.46] vs. AOR: 1.84 [95% CI: 1.36–2.47], respectively).

### HPV persistence

**Figure 2:**
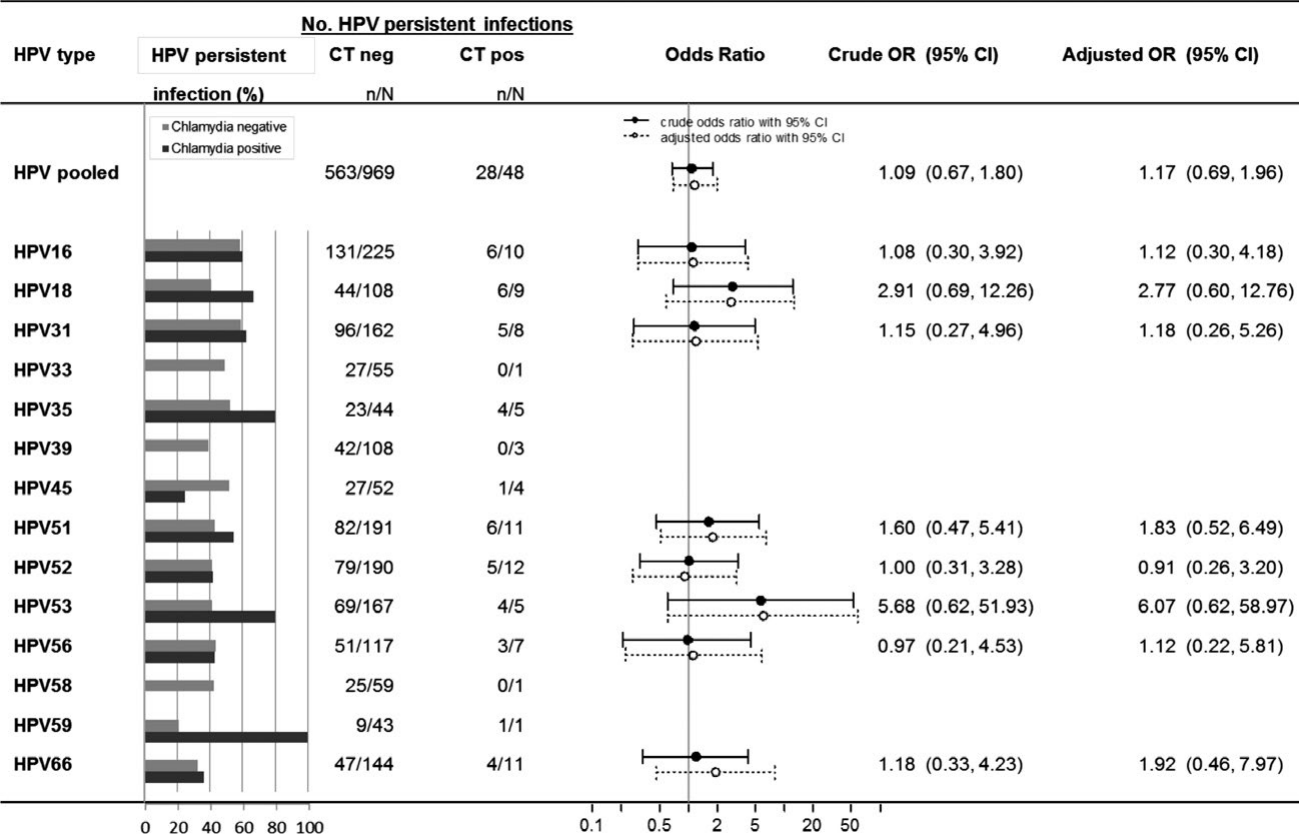
Effect of *Chlamydia trachomatis* (CT) infection at follow-up on type-specific human papillomavirus (HPV) persistence among 1982 Dutch women participating in the Chlamydia Screening Implementation study between 2008–2011. The following data is given for 14 high-risk HPV types, pooled and type-specific, using the imputed data set: number of HPV-persistent cases stratified by CT positivity at follow-up; crude and adjusted odds ratio with 95% confidence intervals. The model was adjusted for number of years being sexually active, number of sexual partners in the last 6 months, condom use with the steady and/or casual partner. The denominator of the pooled estimate is the number of individuals positive for at least one of the 14 HPV types at baseline, the numerator is the number of individuals with at least one persistent infection. For the type-specific estimates the denominator is the number of individuals positive for the specific HPV type at baseline, the numerator is the number of individuals with a persistent infection for the specific HPV type.

The 1-year persistence rate ranged from 22.7% for HPV59 to 59.4% for HPV31 over the follow-up period (Table2). The average follow-up time for women with a persistent HPV infection did not differ from that of the total study population (i.e., 349 vs. 352 days, respectively). The five HPV types with the highest 1-year persistence rates were HPV16 (58.3%), HPV31 (59.4%), HPV33 (48.2%), HPV35 (55.1%), and HPV45 (50.0%). When stratifying for chlamydia co-infection, differences in HPV persistence between chlamydia-infected and noninfected women were not as clear and consistent as for HPV incidence. Pooled persistence rates for those infected and noninfected were 58.3% (95% CI: 44.3–71.2) and 58.1% (95% CI: 55.0–61.2), respectively. However, the numbers of chlamydia-infected women who were HPV-positive at baseline were small, resulting in wide confidence intervals in the type-specific analyses. To obtain robust estimates in subsequent analyses, we selected only HPV types represented by at least five HPV-positive women with chlamydia co-infection: HPV16, -18, -31, -51, -52, -53, -56, and -66. For incident HPV infections the rates were clearly higher for chlamydia-infected women than for chlamydia-uninfected women, whereas the pattern was less distinct for persistence rates (Fig.2). The pooled estimate for the eight HPV types was not significant (AOR: 1.23 [95% CI: 0.68–2.23]) but might suggest a small effect of chlamydia on HPV persistence. When assessing the effect of chlamydia on HPV type-specific persistence, the largest effects were observed for HPV18 and HPV53 (AOR: 2.77 [95% CI: 0.60–12.76] and AOR: 6.07 [95% CI: 0.62–58.97], respectively). The ORs of chlamydia with the various HPV types did not differ significantly from one another (*P *=* *0.80). Results from analyses of complete cases and imputed data are shown in Figure S2.

When assessing the effect of a baseline chlamydia infection on 1-year HPV persistence, an AOR below one (AOR: 0.62 [95% CI: 0.41–0.94]) was found. This suggests that women with a chlamydia infection at baseline have less chance of persistent HPV infection than women without a chlamydia infection at baseline.

## Discussion

In a large follow-up study of young and sexually active women, we assessed the possible association between chlamydia infection and the subsequent acquisition and persistence of infection with carcinogenic HPV types. We found that HPV incidence rates were significantly higher among women with chlamydia co-infection as compared to those without. However, chlamydia co-infection had no significant effect on clearance of pre-existing HPV infections in our analyses.

Our study is unique in that we analyzed the associations between chlamydia and HPV incidence and persistence in a type-specific manner. However, we could not confirm whether associations between chlamydia and HPV differ among types. This negative finding could reflect insufficient statistical power (as the study was not designed to detect type-specific differences) or indicate a truly uniform effect of chlamydia co-infection on the acquisition and persistence of carcinogenic HPV types. The effect of chlamydia on HPV incidence was studied in substantially more women than its effect on HPV persistence. Still, the relative risks of HPV infection among chlamydia-positive versus -negative women showed little variation across HPV types, suggesting that type-specific deviations from the overall association between chlamydia and HPV acquisition are small. A side note to be made is that it could be possible that some of the infections defined as incident infections are in reality latent infections which reactivated again.

A possible biological explanation for the increased risk of HPV infection among women with chlamydia co-infection is that chlamydia causes local inflammation leading to damage of the epithelial tissue which could make a woman more susceptible for HPV infection, irrespective of HPV type 21. Sex-related behaviors offer an alternative explanation for the positive association between chlamydia and HPV acquisition, as chlamydia and HPV both are STIs. To make optimal use of our data and avoid deleting information during multivariate analyses, we imputed information on sexual risk behavior that was missing from our questionnaires. The imputed results differed little from the results for the complete cases. After correction for sexual risk factors, the ORs for the effect of a chlamydia infection on overall and type-specific HPV incidence came slightly closer to one, indicating that behaviors influence the association between the two infections. It is possible that, besides the sexual factors we included, others play a role.

Lack of power is manifest in our analysis of type-specific 1-year HPV persistence, as we observed sizeable differences among relatively small numbers regarding the effect of chlamydia co-infection on clearance of pre-existing HPV infection across types. Moreover, the follow-up that we used may have been too short to detect an increased persistence of HPV infections among chlamydia-positive as compared to chlamydia-negative women as our sampling interval was of the same order as the natural duration of HPV infection 10. Interestingly, we found AORs of around 2 and higher for HPV18, -51, -53, -66, which have a relatively short clearance period (on average 7–10 months). In contrast, AORs around 1 were found for HPV16, -31, -52, -56, for which clearance takes longer (on average 11–14 months). Median follow-up in our study was 11 months, which may have been insufficient to detect increased persistence for some HPV types. Notice that those HPV types with low natural clearance rates also happened to be relatively common at baseline, further contributing to a potential lack of power in assessing the effect of chlamydia on HPV persistence.

Our interpretation is consistent with Shew et al., who looked at the effect of chlamydia on the duration of HPV persistence 4. Collecting samples every 3 months for 3 years, they found that women with a chlamydia infection had a slower overall HPV clearance; however, no HPV type-specific analyses were performed. Few studies have analyzed the effect of chlamydia on HPV persistence 3–5. Silins et al. randomly selected women who had tested HPV-DNA-positive in a population-based HPV screening trial. At follow-up, on average 19 months later, women were retested for HPV, and serum was tested for chlamydia antibodies. An association was found between a self-reported chlamydia infection and carcinogenic HPV persistence but no significant effect was seen for a positive chlamydia test, after correcting for several confounding factors 5. Although these results are consistent with ours, testing chlamydia in serum is not fully comparable with its testing in a genital swab: chlamydia antibodies in serum also include a past infection, whereas chlamydia DNA in a vaginal swab indicates a current infection 22. Samoff et al. assessed the effect of chlamydia co-infection on HPV persistence in adolescent girls 3. Subjects were enrolled at a primary care clinic, where a cervical swab or urine sample was collected to test for HPV-DNA and chlamydia. At follow-up 6 months later, participants were retested for both infections. As in our study, the authors could not confirm an effect of chlamydia at follow-up with respect to persistence of carcinogenic HPV infections. However, they did find a significant positive effect of a concurrent chlamydia infection at baseline on carcinogenic HPV persistence. This result is in contrast to our study. We found that having a chlamydia co-infection at baseline results in less persistent HPV infections (i.e., OR below one), whereas having a chlamydia co-infection at follow-up suggests more HPV-persistent infections (i.e., OR above one), although the difference was not significant. Our results regarding chlamydia at follow-up lie more consistent of expectation than those regarding chlamydia at baseline. A chlamydia infection detected at baseline would have a short infection period (assuming its clearance shortly after treatment), whereas an infection detected at follow-up could have been present for a few weeks up to a year. However, Samoff et al. also treated baseline chlamydia infections, putting our assumption about treatment into question.

Further research is needed into the relation between chlamydia and type-specific HPV persistence. In prospective studies like ours, a detected chlamydia infection is always treated, limiting its duration. In a recent publication, Seraceni et al. used molecular techniques to distinguish between recently acquired and chronic chlamydia infections in a cross-sectional study design 23. They reported a significantly higher frequency of HPV co-infection, as well as a significantly higher number of HPV types, in chronic carriers of *C. trachomatis*. If a long-lasting chlamydia infection is needed to hamper clearance of an HPV infection, our intervention study may have been too short to observe an effect of chlamydia co-infection on HPV clearance. The optimal next step would be the retrospective testing of half-yearly samples from a longitudinal study, with sufficient length of follow-up. Using samples already collected for other purposes would solve the ethical problem of including long-lasting chlamydia infections.

In conclusion, chlamydia is positively associated with an incident infection with a carcinogenic HPV type. This could reflect enhancement in HPV acquisition by chlamydia or residual confounding through unobserved risk factors. Although no significant association was found for chlamydia and HPV persistence, our results point toward an increased persistence of carcinogenic HPV types in the presence of chlamydia co-infection. Our study was not designed to assess possible interactions between chlamydia and HPV, which may have hampered the strength of some of the findings. However, the results do give more than sufficient reason to explore more fully the association between chlamydia and HPV type-specific persistence.

## Supporting Information

**Figure S1.** Effect of a chlamydia infection at follow-up on type-specific HPV incidence among 1982 Dutch women participating in the Chlamydia Screening Implementation study between 2008 and 2011. The crude and adjusted odds ratio with 95% confidence intervals is given for 14 high-risk HPV types, pooled and type-specific, using the complete cases (left figure) and the imputed data (right figure). The models were adjusted for number of years being sexually active, number of sexual partners in the last 6 months, condom use with the steady and/or casual partner. The denominator of the pooled estimate is the number of individuals negative for at least one of the 14 HPV types at baseline, the numerator is the number of individuals with at least one incident infection. For the type-specific estimates the denominator is the number of individuals negative for the specific HPV type at baseline, the numerator is the number of individuals with an incident infection for the specific HPV type.

**Figure S2.** Effect of chlamydia infection at follow-up on type-specific HPV persistence among 1982 Dutch women participating in the Chlamydia Screening Implementation study between 2008 and 2011. The crude and adjusted odds ratio with 95% confidence intervals is given for 14 high-risk HPV types, pooled and type-specific, using the complete cases (left figure) and the imputed data (right figure). The models were adjusted for number of years being sexually active, number of sexual partners in the last 6 months, condom use with the steady and/or casual partner. The denominator of the pooled estimate is the number of individuals positive for at least one of the 14 HPV types at baseline, the numerator is the number of individuals with at least one persistent infection. For the type-specific estimates the denominator is the number of individuals positive for the specific HPV type at baseline, the numerator is the number of individuals with a persistent infection for the specific HPV type.
