# Supplementary figures and images for: Incidence and persistence of carcinogenic genital human papillomavirus infections in young women with or without Chlamydia trachomatis co-infection
Source: Cancer Med. 2015 Jul 21;4(10):1589–98. doi: 10.1002/cam4.496 (PMC4618629; doi:10.1002/cam4.496)

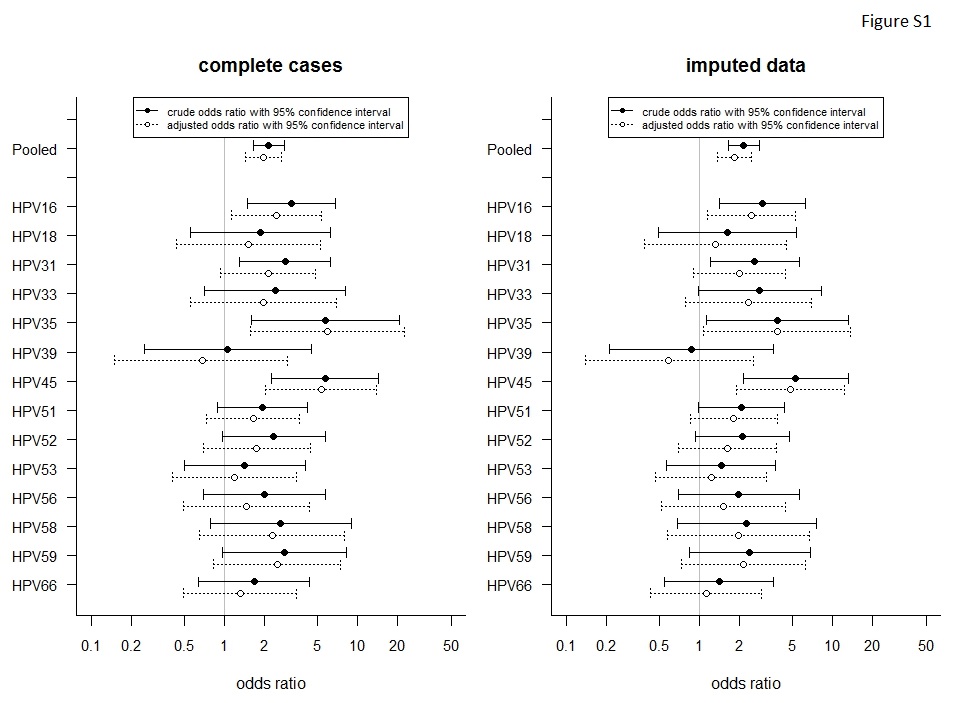

Supplement: Supplementary file 1 [file cam40004-1589-sd1.tif]

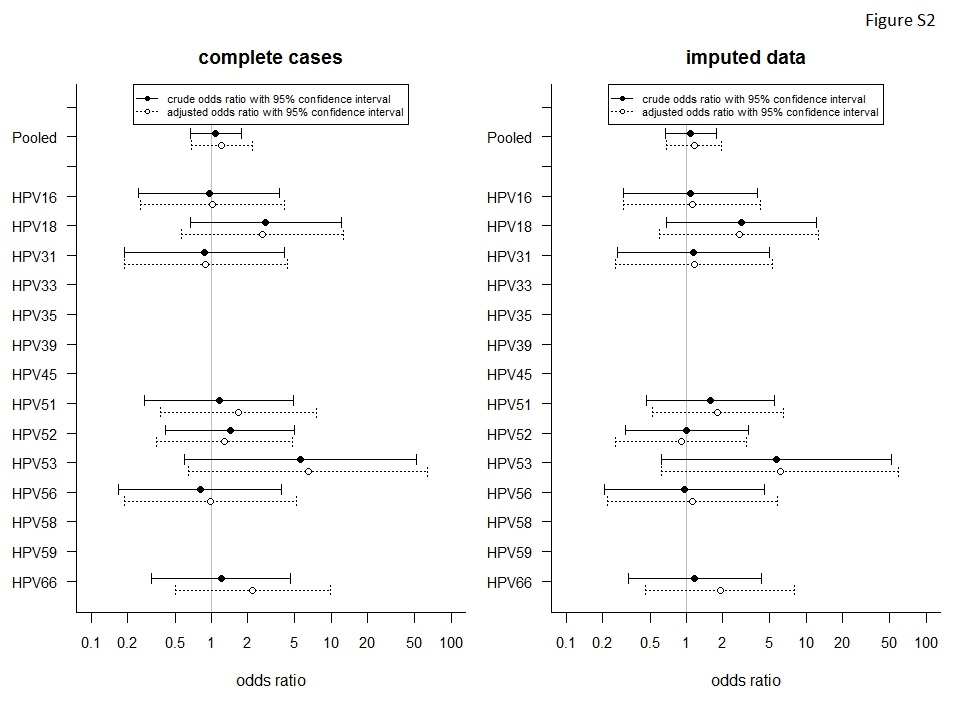

Supplement: Supplementary file 2 [file cam40004-1589-sd2.tif]
